# Surgical management and outcomes of traumatic global brachial plexus injury: A concise review and our center approach

**DOI:** 10.1515/med-2023-0817

**Published:** 2023-10-03

**Authors:** Mohamed Badie Ahmed, Salim Al Lahham, Ghanem Aljassem, Ayman A. H. Asnaf, Zaki T. N. Alyazji, Rand Y. Omari, Fatima Saoud Al-Mohannadi, Abeer Alsherawi, Semir Vranic

**Affiliations:** College of Medicine, QU Health, Qatar University, Doha, Qatar; Plastic Surgery Department, Hamad General Hospital, Hamad Medical Corporation, Doha, Qatar

**Keywords:** brachial plexus, global injury, trauma, nerve transfer, muscle transfer

## Abstract

Global brachial plexus injury (GBPI) mainly affects adults and causes severe life-changing consequences that lead to the deterioration of patients’ quality of life. Several surgical approaches have been described and reported in the literature to improve patients’ functional ability. A literature review is done on PubMed/MEDLINE and Embase using specific keywords to retrieve relevant articles assessing different surgical approaches for GBPI management. Inclusion and exclusion criteria were applied, and eligible articles were included in the review. The literature survey revealed that various surgical options had been used to manage GBPI patients. In this concise review, we discuss and compare the different surgical approaches related to GBPI and its outcome in terms of restoring elbow flexion and extension, shoulder abduction, and wrist and hand function. The primary surgical intervention relies mainly on transferring single or multiple nerves with/without nerve grafts to restore the function of the targeted muscle. Different techniques using a variety of nerve donors and recipients are compared to assess the functional outcomes of each option. Moreover, further options are addressed for delayed GBPI injuries or failed nerve transfer procedures, as in free functional muscle transfer techniques. In addition, information about brachial plexus injury cases faced in our center is presented along with our center’s approach to diagnosing and managing partial and GBPI cases.

## 1 Introduction

Global brachial plexus injuries (GBPI) are life-altering injuries that might lead to physical and psychological impairment and disability. The most common cause of these injuries is trauma, and most affected patients are adults [1]. It has been reported that approximately 1.2% of the patients presenting to a trauma facility suffer from brachial plexus injury, most of whom are young male patients [2]. The brachial plexus might be injured at any level including upper, lower and total, or global injury (Table 1). GBPI is a severe and devastating event where all the plexus is injured, causing a severe functional deficit. Moreover, the nature of the injury limits surgeons due to scanty treatment options and the number of donor nerves. Thus, surgical management aims to restore essential upper limb functions. The highest priority is elbow flexion, followed by shoulder abduction and stability, and wrist and hand functions [3]. Although many surgical techniques have been applied and investigated to assess its outcome and success rate, managing GBPI represents a major challenge in upper limb reconstruction. Nerve transfer is considered the best option in treating brachial plexus injuries compared with other options, such as muscle transfer or tendon transfer. The same applies to GBPI but with fewer nerve donors available for the transfer. Therefore, multiple nerve transfer procedures have been described in GBPI events and their outcomes have been reported to assess the best option required to restore certain functions. In this mini-review, we aim to discuss the surgical options reported in the management of GBPI and its functional outcome.

**Table 1:** Brachial plexus level of injury with their rate

Study	Lemus et al. [23]	Cho et al. [24]
Level of injury	Rate (%)	
Upper injury (mainly C5, C6)	66.67	59
Lower injury (C8, T1)	10.71	8
Total injury C5-T1	22.61	33

## 2 Methods

**Figure 1:**
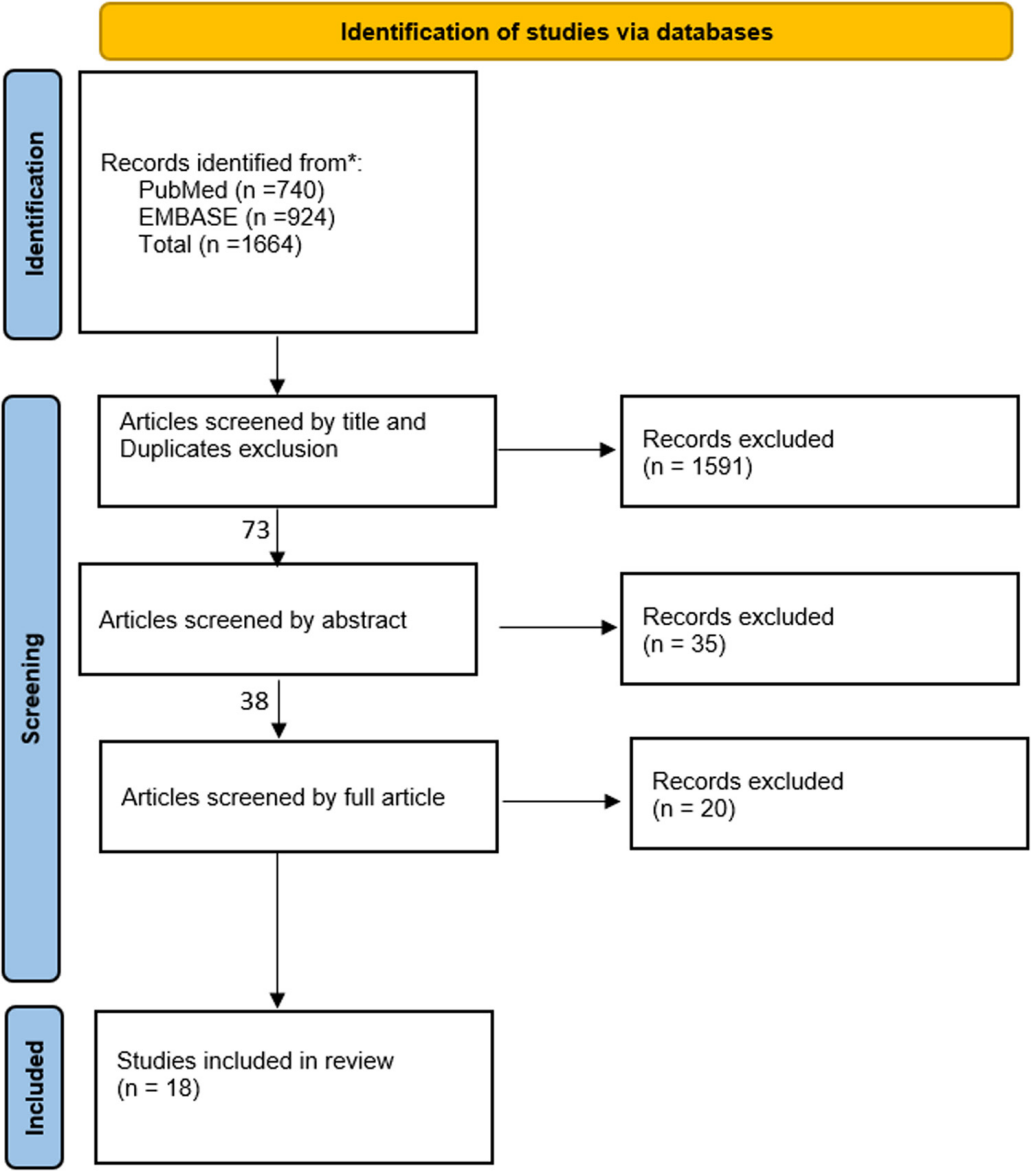
Flow chart of the selected studies.

The literature review was conducted on the available sources that describe surgical management and outcomes of traumatic brachial plexus injuries between 1990 and 2022. The databases used in this review were PubMed/MEDLINE and Embase. The following keywords were used: (surgical management) OR (surgical intervention)) OR (surgical treatment)) AND (trauma)) OR (traumatic)) AND (global brachial plexus injury)) OR (total brachial plexus injury)) OR (global brachial plexus avulsion)) OR (total brachial plexus avulsion)) OR (global brachial plexus palsy)) OR (total brachial plexus palsy)) AND (adults). Articles were classified based on their appropriateness and relevancy for this review. Most studies were excluded based on their titles and abstracts (Figure 1). Then, the final exclusion was based on the full article. Additionally, we performed a manual reference search of retrieved studies. Inclusion criteria were English-written articles, articles that investigate GBPI, trauma as a cause of the injury, articles that target the adult population, articles that discuss surgical management, and articles that assess the surgical outcome based on validated methods. Exclusion criteria were non-English articles, articles that investigate partial brachial plexus injuries, causes of BPI other than trauma, pediatric or geriatric population, articles that discuss non-surgical interventions or diagnosis approach, and articles that did not assess the surgical outcome.

The review is divided into subheadings containing the management and outcomes of each movement based on the reconstruction priority. The last section focuses on the muscle transfer options that could be used for various brachial plexus injuries and are not limited to traumatic global injuries. The information found in the selected studies on surgical management and outcomes of traumatic GBPI was thoroughly evaluated, and it is presented and discussed in the following sections.

## 3 Results

### 3.1 Elbow flexion

Elbow flexion is the first action that surgeons aim to restore as it is a primary movement needed in most daily activities. Thus, several articles discussed different approaches that could be used to reinnervate the muscles responsible for the action. Liu et al. conducted a study comparing phrenic and intercostal nerves’ ability to restore elbow flexion in GBPI patients after their transfer to the anterolateral bundle of the anterior division of the upper trunk anterolateral bundles of the musculocutaneous nerve, respectively [4]. The British Medical Research Council grading system was applied to evaluate the flexion strength in the two groups. The scale ranges from 0 to 5, and a grade of 3 or above was considered effective muscle power. The phrenic group’s effective motor recovery rate (EMRR) was 83% compared with 70% in the intercostal group. Moreover, the outstanding elbow flexion angle rate (>120°) was 48 and 40% in the phrenic and intercostal groups, respectively. However, the prognosis of the phrenic group was better; however, no statistical significance was observed between the groups regarding EMRR and the outstanding elbow flexion angle rate.

Another study compared phrenic nerve transfer with and without nerve graft and its ability to restore elbow flexion [5]. The direct nerve transfer group had an EMRR of 86%, while the nerve graft group had 84%; however, statistical significance was not reached. However, the timing of the surgery (within or more than 4 months) showed a statistically significant difference in muscle motor functional recovery. Moreover, Liu et al. assessed EMRR in biceps after transferring the phrenic nerve, intercostal nerves, or the contralateral seventh cervical nerve to the musculocutaneous nerve and showed an 86% recovery rate [6]. Further studies showed biceps muscle EMRR of 66, 77.7, and 33% when using a spinal accessory, intercostal, and contralateral C7 as donor nerves, respectively [7]. In addition, the proximal C5 stump was grafted in 7 patients and 5 (71%) had effective biceps muscle recovery.

Other options to restore elbow flexion in the case of failure of nerve transfer or unsatisfactory results are reported in the literature. Functioning-free muscle transfer (FFMT) is an effective option in GBPI [8]. Yang et al. explored the functional outcome of transferring free gracilis to reconstruct elbow flexion in GBPI patients [9]. The results showed that elbow flexion muscle power was effective (M3 or above) in 85.7% of the patients, and the average range of motion of elbow flexion was 106.5°. Another study assessed elbow flexion in traumatic brachial plexus injury patients. No difference in elbow flexion outcome was detected when comparing patients with FFMT innervated by either intercostal nerve or spinal accessory nerve grafts [10].

### 3.2 Shoulder abduction

Shoulder stability and abduction is the second most crucial action that should be restored after elbow flexion [3]. Several studies discussed options to restore this function. However, the options in GBPI are sparse due to the severity of the injury and the number of nerves affected. Bhatia et al. conducted a cohort study on patients with GBPI and assessed the role of nerve reconstruction in restoring several functions, including shoulder abduction [7]. Transferring the spinal accessory nerve to the suprascapular nerve showed 88% EMRR of the supraspinatus muscle. Moreover, the abduction angle restored in most cases was 30–45° and reached up to 70–80° in a few cases.

Another study examined the transfer of the spinal accessory nerve or the contralateral lateral thoracic nerve to the suprascapular nerve on supraspinatus muscle recovery [6]. The reported EMRR was 54%. A new technique to restore shoulder stability and abduction was reported in one case with GBPI in the literature [11]. The technique utilizes the contralateral spinal accessory nerve and transfers it to the suprascapular nerve using a nerve graft (sural nerve). The patient regained active shoulder abduction (26°) and external rotation (15°) after 24 months of follow-up after the surgery. The authors proposed this technique as a reliable option for GBPI shoulder reanimation in cases where transferring the ipsilateral spinal accessory nerve is impossible.

### 3.3 Elbow extension

Restoring elbow extension is essential for elbow stability and normal movement without using the contralateral hand to stabilize the elbow. Thus, surgeons started paying attention and considering different techniques to restore elbow extension [12]. Zheng et al. studied the possibility of using phrenic and intercostal nerves to restore elbow flexion and extension in the same patients [13]. The results showed that 85% of the patients had efficient elbow flexion, while none of them had efficient elbow extension. Thus, they recommended avoiding intercostal nerve transfer after phrenic nerve transfer in patients with GBPI.

On the other hand, Gao et al. evaluated the outcome of intercostal nerve transfer to the nerve of the long head of the triceps muscle [14]. The study included two groups, one with two intercostal and three intercostal nerve transfers. The first group (two intercostals) had an EMRR of 55.56%, while the second group had 57.14% with no significant statistical difference. Moreover, the results approved the possibility of transferring intercostal nerves to restore elbow extension even if combined with phrenic nerve transfer to restore elbow flexion, which is opposite to the previous article’s suggestion. In this study, the EMRR of the biceps and triceps muscles in patients with phrenic and intercostal nerve transfer was 88 and 56%, respectively. Therefore, combining phrenic and intercostal nerve transfer is a valid option for patients requiring these surgeries.

Another study compared intercostal nerves with contralateral C7 nerve transfer to the long head branch of the triceps and evaluated elbow extension outcomes [15]. The elbow extension EMRR was 47 and 28.5% in the intercostal and contralateral C7 nerve groups, respectively, with no significant difference. Bhatia et al. assessed triceps function after intercostal nerve transfer and reported grade 2 muscle recovery as a successful outcome since patients cannot abduct shoulders above 90° [7]. As a result, the successful muscle recovery rate was 80%. In contrast, Liu et al. reported efficient triceps muscle recovery after nerve transfer as grade 3 or above, and the EMRR was 46% [6].

### 3.4 Hand and wrist functions

Restoring hand and wrist functions is not a priority in GBPI patients; elbow flexion and shoulder stability are at the top. However, restoring their functions improves patients’ satisfaction and quality of life. One study assessed the efficacy of transferring contralateral C7 to different recipient groups (median nerve, median nerve + biceps branch, median nerve + triceps branch) [16]. Flexor carpi radialis EMRR was 57.7% in the median nerve, 45.5% in the median nerve + biceps branch, and 36.4% in the median nerve + triceps branch groups, with no significant difference. Moreover, wrist and digital flexion range of motions significantly improved across the three groups after the surgery. In cases of two nerve transfers, the biceps branch showed a better outcome than the triceps branch. Thus, the authors recommended the use of the biceps branch with a median nerve in the transfer.

Another study evaluated the possibility of restoring wrist and intrinsic muscle flexion using a nerve graft for the median nerve from ipsilateral proximal nerve root stumps or contralateral C7 [7]. The first group results showed that 27% of the patients had grade 2 muscle recovery, while finger flexion was achieved in 26% of the second group. In addition, intercostal nerves and contralateral C7 nerve were transferred to the median nerve, and the outcome was reported by Liu et al. Finger flexor muscles showed an EMRR of 43% [6].

### 3.5 Muscle transfer options

Muscle transfers are an optimal approach to restore many upper limb functions in cases of delayed GBPI reconstruction or failure of nerve transfer options.

Free gracilis muscle transfer was used in the late 1990s to restore hand functions and resulted in a good outcome [17]. It was also used to restore elbow flexion and finger extension. However, the outcome was less effective when compared with the combined gracilis adductor longus triple free functioning muscle transfer, described by Sananpanich et al. [18]. This technique outcome showed satisfactory finger extension and good hook grip of the hand with good elbow flexion [19].

One study assessed the functional outcome of GBPI patients managed by nerve transfer, single-free muscle, or double-free muscle transfer [20]. The results showed no difference in shoulder abduction and flexion among the three groups. However, the shoulder’s external rotation and the elbow flexion range were significantly higher in DFMT patients.

Using double-free functioning muscle transfer to restore elbow flexion and hand functions was also reported by Doi et al. [21]. The study outcomes showed the ability to restore excellent to good elbow flexion in 96% of the treated patients. In addition, 65% restored >30° of total active finger motion with the second muscle transfer. Moreover, Dodakundi et al. reported a significant improvement in the arm, shoulder, and hand scores after double-free functioning muscle transfer in traumatic total brachial plexus palsy patients [22].

### 3.6 Our center approach

The cases we encounter at our center are closed (90%) or open injuries (10%). Most open injuries are due to penetrating injuries affecting the whole plexus. This type is usually explored and repaired immediately. The closed type is usually secondary to road traffic accidents or direct trauma to the shoulder and is managed conservatively at the beginning. After comprehensive clinical assessment and determining the level of brachial plexus injury, we perform magnetic resonance imaging (MRI) and electromyography (EMG) over 3 weeks as a baseline investigation. Then, another set of investigations is done 3 months postinjury if needed. Patients are referred to occupational therapy and physical rehabilitation to prevent any muscle atrophy or contracture deformity and to prepare them for surgical interventions if required. At 3 months, if there is no recovery of elbow flexion or a paradoxical recovery pattern is observed, new EMG and MRI are requested to accurately determine the type and level of brachial plexus injury. Then, the surgical intervention will be planned based on the clinical assessment and investigation outcome.

**Figure 2:**
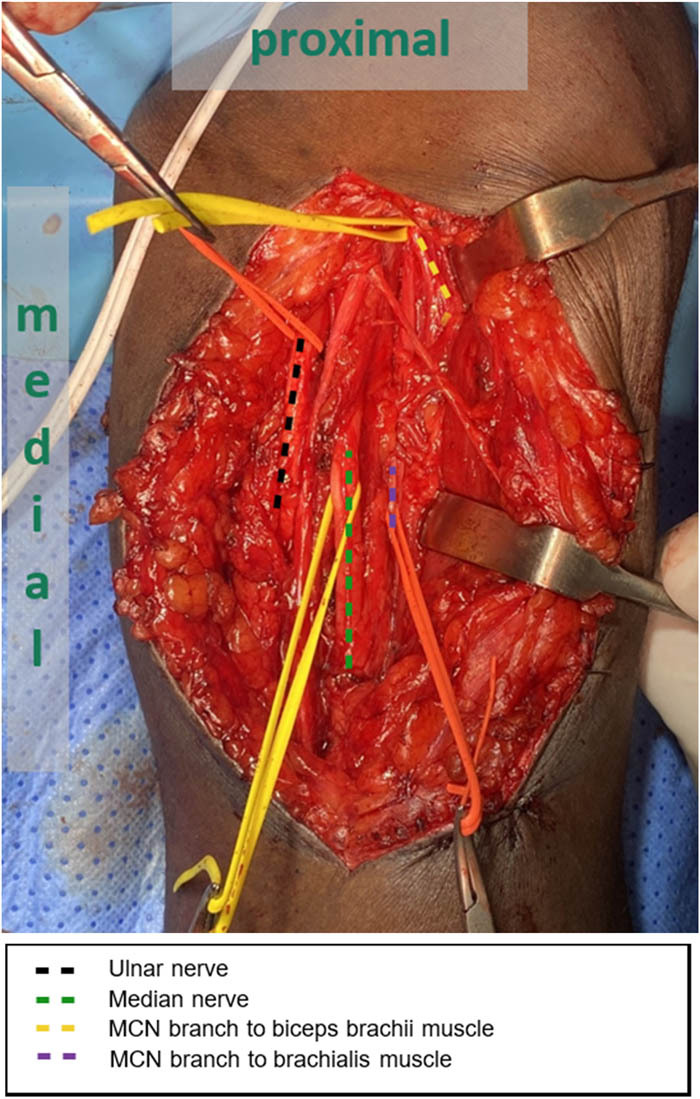
Exploration findings of brachial plexus peripheral nerves through a medial arm incision. The biceps muscle is retracted to the lateral side.

**Figure 3:**
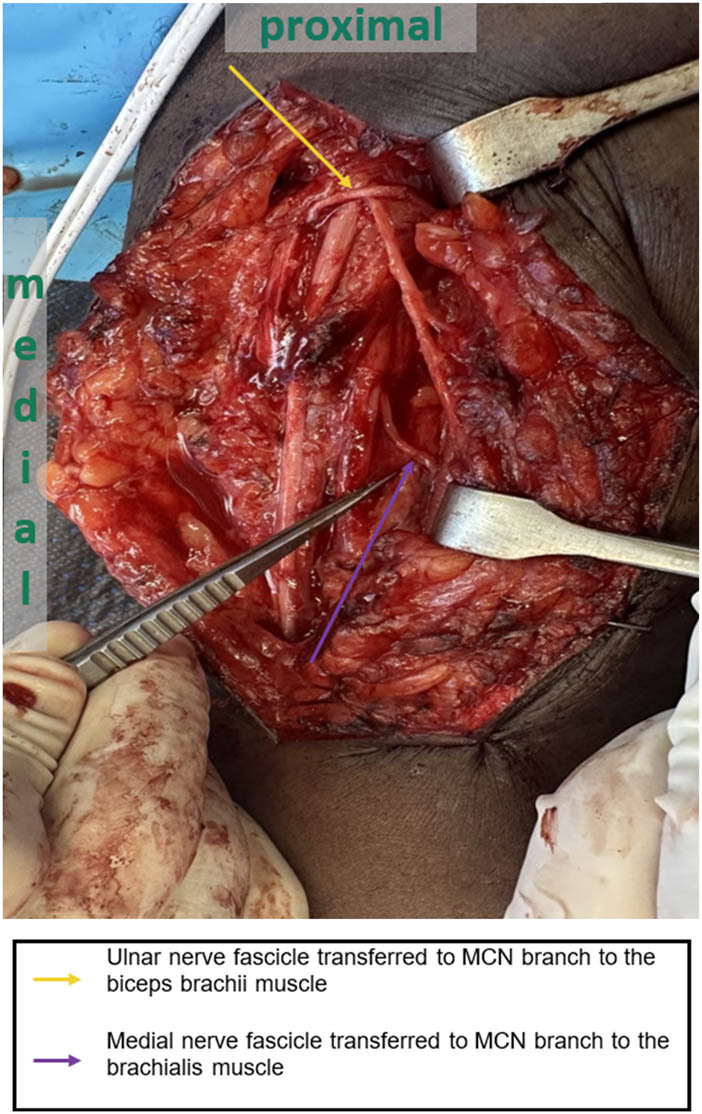
Transfer of one of the fascicles that innervate FCU muscle from the ulnar nerve to the MCN branch of the biceps brachii muscle. Transfer of one of the fascicles that innervate the FCR muscle from the median nerve to the MCN branch of the brachialis muscle. The choice of the fascicles was confirmed intra-operatively using a nerve stimulator.

Modified Oberlin transfer is usually conducted in case of lower roots recovery with no signs of elbow flexion (Figures 2 and 3). However, if GBPI is confirmed, the following procedures are usually done. Spinal accessory nerve transfer to the suprascapular nerve is done to restore shoulder function. After that, elbow flexion is restored after transfer of intercostal nerves through free innervated muscle (mainly gracilis muscle). Then, wrist fusion is done, followed by elongation of the free innervated muscle using fascia lata for finger flexion and elbow flexion simultaneously. In patients with supination fixed deformity, rotational osteotomy is done to correct the forearm posture into a mid-pronation position for functional outcome. The recovery period might take up to 2 years, where patients are followed at 2 weeks postoperative and then after 1 month. After that, follow-up appointments are given every 3 months. EMG can be done after 3 months of nerve transfer to check for any signs of reinnervation and can be repeated at 6 months postoperative if the clinical assessment shows progression.

## 4 Conclusions

GBPI is a devastating event that can lead to major restrictions and a severe decline in patients’ quality of life. Also, the nature of the injury limits many surgical options that could be used to manage these cases. Several surgical managements have been suggested to restore the most important functions that may improve patients’ ability to perform essential daily activities independently. However, the functional outcome varies across studies due to several factors. Based on our center’s experience, managing GBPI patients is very challenging. The management should address several factors, including feasible surgical options, patient priorities and needs, and follow-up system availability. More extensive studies should be conducted to validate the optimal surgical options for different GBPI cases.
